# Are Cancer Patients' Socioeconomic and Cultural Factors Associated with Contact to General Practitioners in the Last Phase of Life?

**DOI:** 10.1155/2015/952314

**Published:** 2015-08-27

**Authors:** M. A. Neergaard, F. Olesen, J. Sondergaard, P. Vedsted, A. B. Jensen

**Affiliations:** ^1^The Palliative Team, Aarhus University Hospital, Noerrebrogade 44, 8000 Aarhus C, Denmark; ^2^The Research Unit for General Practice, Aarhus University, 8000 Aarhus C, Denmark; ^3^The Research Unit for General Practice, University of Southern Denmark, 7000 Odense, Denmark; ^4^Research Centre for Cancer Diagnosis in Primary Care, The Research Unit for General Practice, Aarhus University, 8000 Aarhus C, Denmark; ^5^Department of Oncology, Aarhus University Hospital, 8000 Aarhus C, Denmark

## Abstract

*Introduction.* General practitioners (GPs) play an important role in end of life care, which should be offered regardless of socioeconomic position and cultural factors. The aim was to analyse associations between GP contacts at the end of life and socioeconomic and cultural characteristics of Danish cancer patients. *Method.* Population-based study identifying 599 adults who died of cancer from March to November 2006, in Aarhus County, Denmark. Associations between health register-based data on “total GP face-to-face contacts” and “GP home visits” during the last 90 days of life and patients' socioeconomic and cultural characteristics were calculated. *Results.* Having low income (RR: 1.18 (95% CI: 1.03; 1.35)) and being immigrants or descendants of immigrants (RR: 1.17 (95% CI: 1.02; 1.35)) were associated with GP face-to-face contacts. However, patients living in large municipalities had lower likelihood of having both GP face-to-face contacts in general (RR: 0.85 (95% CI: 0.77;0.95)) and GP home visits (RR: 0.89 (95% CI: 0.80; 0.99)). *Conclusion.* This study indicates higher proportion of GP contacts to economically deprived patients and immigrants/descendants of immigrants. These subgroups were, however, small and results should be looked upon with caution. Furthermore, palliative needs were not included and together with urban/rural the underlying causes need further investigation.

## 1. Introduction

General practitioners (GPs) play an important role in the end of cancer patients' lives [1, 2]. For example, GP home visits in the last months of life seem to facilitate possibilities of dying at home [3, 4] where most terminally ill cancer patients prefer to die and to spend as much time as possible [5–8].

Since the Danish health care system is tax financed, Denmark (DK) is often referred to as a country with a high level of equality in health care [9]. However, in a recent study we found that dying at home was negatively associated with having a middle personal income compared with a high income [10]. We also found an overall equality in access to specialised palliative care (SPC) and a higher likelihood of access to SPC among immigrants and descendants of immigrants [11]. However, only a few studies have investigated GPs' services at the end of life in relation to socioeconomic and cultural factors. Three Canadian and one UK study have shown mixed results concerning socioeconomics and GP services [12–15]. In these studies income areas, patients' self-reported financial strain, and household income, respectively, were used to evaluate patients' economic status. None of the studies included ethnicity. Hence, there is a need for investigating inequality with high quality person-based register data in care in the last phase of life in relation to GP involvement.

The aim of this paper was to analyse associations between GP contacts (face-to-face contacts in general and home visits, resp.) in the last phase of life and socioeconomic and cultural characteristics of Danish patients, who died of cancer.

## 2. Materials and Methods

This study is a population-based register study carried out in the former Aarhus County, DK.

### 2.1. Setting

At the time of the study, Aarhus County comprised approximately 640 000 inhabitants (equivalent to 12% of the Danish population) and 43 municipalities.

Danish citizens receive free, tax-financed medical care. GPs are responsible for frontline care 24 hours a day with a large GP cooperation providing out-of-hours care (4PM–8AM on weekdays and all day on bank holidays) [16]. Community nurses may visit patients in their homes on a 24-hour basis. GPs can ask for advice or refer patients to outgoing SPC hospital-based teams, if symptom relief or problem-solving is complex.

### 2.2. Sampling of Cohort

**Figure 1:**
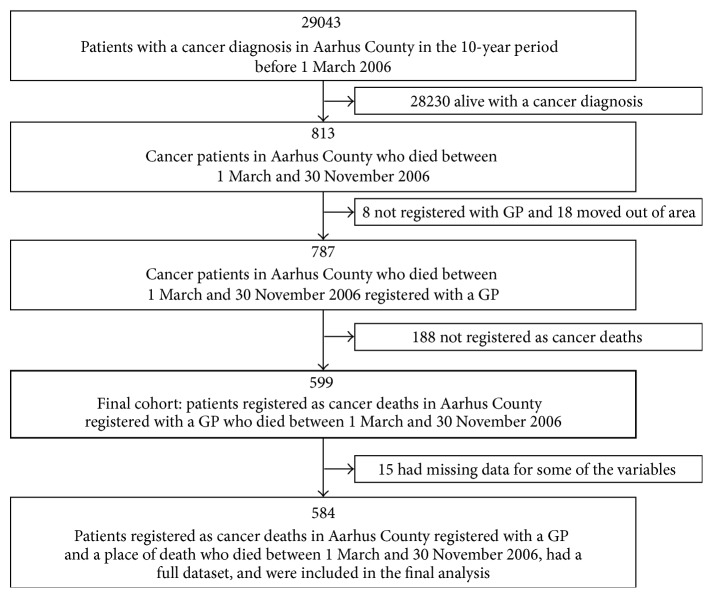
Flowchart for sampling of study population and inclusion of cases for further analysis. GP: general practitioner.

The cohort included 599 adults (≥18 years) who died from cancer in the period from 1 March to 30 November 2006 in Aarhus County. These nine months were originally chosen since the bereaved relatives should have a chance to receive a questionnaire within a certain time-frame from the patients' death [17]. The dataset came from four health registers: the county hospital discharge register, the Danish Civil Registration System, the regional health authority's register, and the Danish Register of Causes of Death (Figure 1). This was possible since Danish residents since 1968 have been assigned a unique 10-digit personal identification number. The cohort is also described in previous studies [10, 11].

### 2.3. Register Data

Additional data were subsequently collected through the central government agency of statistics in DK (*Statistics Denmark*), and access was achieved via the Research Centre for Cancer Diagnosis in Primary Care, Aarhus University. Registers used are described in prior studies [7, 10, 11].

Data were retrieved on patient-related factors at the end of 2005: the patient's marital status (single, married/cohabiting) and having children living at home (no/yes). The patient's personal yearly income was divided into three groups (0–10,000 English Pounds (£)/year, 10,001–20,000 £/year, and >20,000 £/year). Employment was divided into three groups: (1) unemployed, on social security and student; (2) old age or early retirement pensioner; or (3) employed or leadership position. Furthermore, we retrieved data on ethnicity (immigrant/descendant, not immigrant/descendant) and degree of urbanicity (<10,000, 10,000–49,999, 50,000–99,999, and ≥100,000 inhabitants in the municipality).

Concerning use of health care services, we included these services in the last 90 days prior to death of the patient since prior studies have found that care in this period is important to, for example, place of death [3, 4, 18]. We retrieved data on the face-to-face contacts with the GP (home visits and other face-to-face contacts: in-office consultations and conversational therapy) in the last 90 days of life. Furthermore, as we wanted to adjust for other professional contacts we enclosed data on days spent at hospital (continuous variable) and whether a specialist palliative care team had been involved in up to two years prior to death (no, yes). Unfortunately, contacts with community nurses are not registered in national registers in Denmark.

### 2.4. Analysis

In the uni- and multivariate analysis “total GP face-to-face contacts” and “GP home visits” during the last 90 days of life were defined as outcome measures and associations with the patient's socioeconomic and cultural characteristics were calculated.

Data on total GP face-to-face contact (i.e., in-practice consultations and home visits) were grouped into 0 or 1 contact and ≥2 contacts and GP home visits were grouped into yes/no. These cut-offs were chosen, since only one face-to-face contact could not be seen as involvement, whereas even a single home visit by the GP has been shown to make a clinical difference [3, 4].

We adjusted for gender, age at the time of death (continuous variable), and cancer diagnosis (lung, colon/rectum, prostate, breast, and other). Since days spent in hospital in the last 90 days of the patient's life (continuous variable), place of death (home death (including nursing home)/institutional death), and “involvement from a specialist team” (yes/no) may be associated with both GP involvement and social position, these variables were included as confounders.

All variables in the multivariate model were assessed for collinearity (Pearson's correlation coefficient <0.4) and multicollinearity (variance inflation factor (VIF) <10) [19]. Employment was withdrawn from the model because of a strong collinearity with income.

Some variables had missing values (educational level:* n* = 70, economic status:* n* = 7, marital status and having children living at home:* n* = 1, and place of death* n* = 7). Data on education were missing especially among older patients, because this variable was not systematically registered in DK before 1973. Therefore, we did not include this variable in the final model. Finally, 584 patients were available with full dataset for further analysis.

Since there was a high prevalence of outcome measures, odds ratios could tend to overestimate the association [20]. Risk ratios (RRs) with 95% confidence intervals (95% CI) were therefore used and calculated with generalised linear models (GLMs) with log link (the Bernoulli family). The Poisson regression model was used in the adjusted analysis since the model could not converge with GLM [20]. We adjusted for clustering of patients in general practices [21]. Data were analysed using STATA 11.

## 3. Results

Table 1 shows descriptive data for the 584 patients included in the final analysis. The 15 patients left out because of missing values have statistically significantly fewer home visits than patients included in the analysis, but no other significant differences were seen.

### 3.1. Associations with “GP Face-to-Face Contacts in All”

Unadjusted and adjusted analyses of associations with ≥2 GP face-to-face contacts in the last 90 days of the patients' lives are shown in Table 2. It shows that patients with “very low income” (<10,000 £/year) (RR: 1.18 (95% CI: 1.03; 1.35)) and immigrants or descendants of immigrants (RR: 1.17 (95% CI: 1.02; 1.35)) have a higher probability of having GP face-to-face contacts than patients with “normal income” (>20,000 £/year) and patients of Danish origin. Furthermore, patients living in large municipalities (≥100,000 inhabitants) have a lower likelihood of having GP face-to-face contacts than patients living in municipalities with less than 10,000 inhabitants (RR: 0.85 (95% CI: 0.77; 0.95)).

### 3.2. Associations with “GP Home Visits”

Associations between socioeconomics and home visits are shown in Table 2. The adjusted analyses show that patients living in large municipalities (≥100,000 inhabitants) have a lower likelihood of having home visits from their GP than patients living in municipalities with less than 10,000 inhabitants (RR: 0.89 (95% CI: 0.80; 0.99)).

## 4. Discussion

### 4.1. Main Findings

Having a very low income and being immigrant or descendant of immigrants were associated with a higher likelihood of having GP face-to-face contacts in the last 90 days of cancer patients' lives. Furthermore, living in a municipality with a large number of inhabitants was associated with receiving less GP face-to-face contacts and GP home visits.

### 4.2. Strengths and Limitations of the Study

Strengths of this study are the population-based approach and use of comprehensive, high quality register data. Furthermore, patients were sampled from registers, which kept selection bias at a minimum. However, the proportion of immigrants or descendants of immigrants in the population was low leading to low statistical precision concerning association between ethnicity and GP services.

Using register data gave us the possibility of retrieving valid and specific data regarding patients' income. This is a more valid measure than self-reported income or income area. However, we used the year before death to measure socioeconomic factors, which may have been altered by the cancer trajectory. Although this was the same for all groups, it may still be a potential bias in relation to the exact income. Furthermore, the patients died in 2006, and GP services may have changed since then. However, this study addresses the factors associated with GP contacts, and we have no reason to believe that these associations have changed over time.

Using only registers provided no possibility of adjusting for patient-based factors like palliative needs, which are important when investigating inequity. Adjusting for needs may have altered our results, since especially psychological needs may correlate with socioeconomic factors.

### 4.3. Comparison with Existing Literature

Unlike our findings two Canadian studies found that terminally ill patients living in lower income areas were less likely to receive physician home visits compared to patients in higher income area [12, 13]. Cunningham et al. measured income-related inequities in expenditures on services in their last year of life in Canada. They found that health care expenses on GPs showed an inverse correlation with a likelihood of coming from a low income household [15]. The differences between DK and Canada may be explained by different financial structure of health care where GP services are totally free of charge in DK. Also a UK study found that terminally ill patients who felt that they were having financial difficulties were more likely to be frequent attenders in the GP surgeries in the last phase of life [14]. The studies from Canada and UK did not have access to information about patient's personal income and data can therefore not be directly compared to our study.

Access to GP services is driven by at least two factors: patients' request for contact and GPs' actively seeking out patients. Our findings may indicate that financially deprived patients have more palliative care needs or more comorbidity, and they therefore need more health care services. The results may also indicate that GPs stratify their services according to economic position, since this may act as a proxy for frailty in care in the last period of life. It might also be explained by a higher use of other health care services among patients with higher socioeconomic status, but in our final model we adjusted for days spent in hospitals and access to SPC.

We saw that immigrants or descendants of immigrants had more GP contacts in the last three months of life compared to ethnic Danes even when adjusted for socioeconomic factors. However, this result must be looked upon with caution since this group is very small in our population. This association has not been explored before, but earlier studies indicate the same trend for health care in general [22, 23]. One may think that these groups do not have the same access to specialised care and therefore require more care in the primary care sector. However, this does not seem to be the case in DK, since we also found a higher use among immigrants/descendants concerning specialised palliative care [11]. It is also interesting to see that the positive association between ethnicity and GP service is not present when it comes to home visits (Table 2). Although nonsignificant it seems that nonethnic Danes get less home visits from their GP in the last period of life compared to ethnic Danish patients. The reason for a different balance between in-office consultations and home visits among the two groups is probably multifaceted including cultural issues and traditions among both families and doctors. Because of the low number in this group in our study, these findings need further investigation.

Urbanicity seems to make a difference in our study, since living in larger municipalities was associated with fewer GP contacts also after adjusting for days spent at hospital and contact to palliative specialist teams. This could be due to either cultural differences between rural and nonrural areas or structural differences according to organisation of care, access, and treatment. Patients may tend to use their GP less when they have a hospital nearby even though their access to SPC did not differ [11]. Hence, the tradition for contacting the GP may be different in urban areas and the GPs' attitudes towards palliative care and to their own role may also be different. Earlier studies on GP services in general health care in Europe also found that rural general practices provided more comprehensive services regardless of the health care system [24] and an Australian study concluded that GPs in rural areas played greater roles in care coordination, clinical and psychosocial care in colorectal cancer trajectories than GPs in urban areas [25]. But whether these differences are because of patients' different needs, culture in seeking doctors, doctors' attitude towards continuity and commitment, differences in primary care teamwork, or merely an expression for lack of access to hospital care needs further investigation [26].

### 4.4. Implications

With this study we dispose earlier findings of prorich care concerning GP service in the last phase of life, when it comes to a health care system with free access to these services as in DK. However, we found cultural differences, both ethnic and urban/rural, which need further elaboration. However, one has to remember that quality of care does not necessarily correlate with number of contacts and further studies may reveal the type and quality of services and care is contained in the contacts in the last phase of life. The interaction between patient, relatives, GPs, and other health professionals is complex and a focus on socioeconomic and cultural differences in future studies is needed to qualify and further explore the found differences in this study that merely focused on the number of GP contacts.

## 5. Conclusion

The study indicates a higher proportion of GP face-to-face contacts in the last phase of life among financially deprived patients and immigrants/descendants of immigrants. These subgroups were, however, a small part of the population and results should be looked upon with caution. This study did not include palliative needs, which may follow both economic status and ethnicity. Hence, focusing on how to adjust for needs in palliative care in register studies in the future is warranted. Furthermore, the study indicates differences concerning urbanicity and GP contacts in the last phase of life, where the underlying causes need further elaboration.

## Figures and Tables

**Table 1:** Characteristics of the 584 deceased cancer patients included in the analysis. Data derived from Danish health registers.

Sociodemographic characteristics and health utilisation	In all
	(*N* = 584)
Age of patient at time of death (mean years (95% CI))	70.8 (69.8; 71.8)
Age of patient at time of death (*n* (%))	
18–59	119 (20.4)
60–74	236 (40.4)
75+	229 (39.2)
Patient gender (*n* (%))	
Female	261 (44.7)
Male	323 (55.3)
Cancer diagnosis (*n* (%))	
Bronchus/lung	111 (19.0)
Colon/rectum	90 (15.4)
Prostate	78 (13.4)
Breast	65 (11.1)
Others	240 (41.1)
Patient's marital status (*n* (%))	
Single	258 (44.2)
Married or cohabiting	326 (55.8)
Having children living at home (*n* (%))	
No	551 (94.4)
Yes	33 (5.7)
Patient's economic status (*n* (%))	
High income (>20000 £/year)	219 (37.5)
Medium income (10001–20000 £/year)	330 (56.5)
Low income (0–10000 £/year)	35 (6.0)
Patient's highest educational level (*n* (%))	
Primary school	259 (50.4)
Vocational training	164 (31.9)
GCSE	8 (1.6)
Further education (1–4 years after GCSE)	61 (11.9)
Higher education (>4 years after GCSE)	22 (4.3)
Employment (*n* (%))	
Unemployed, social security, student	79 (13.7)
Old age pensioner, early retirement pensioner	399 (69.3)
Employed or leadership position	98 (17.0)
Immigrant/nonimmigrant (*n* (%))	
Not immigrant or descendant	563 (96.4)
Immigrant or descendant	21 (3.6)
Urbanity, inhabitants in municipality (*n* (%))	
10,000 inhabitants	105 (18.0)
10,000–49,999 inhabitants	137 (23.5)
50,000–99,999 inhabitants	111 (19.0)
≥100,000 inhabitants	231 (39.6)
Place of death (*n* (%))	
Home	194 (33.2)
Nursing home	138 (23.6)
Hospital or hospice	252 (43.2)
GP face-to-face contacts^*∗*^ (*n* (%))	
0 contacts	38 (6.5)
1 contact	66 (11.3)
2 contacts	64 (11.0)
3 contacts	81 (13.9)
4 contacts	88 (15.1)
5 contacts	53 (9.1)
≥6 contacts	194 (33.2)
GP home visits^*∗*^ (*n* (%))	
0 home visits	128 (21.9)
1 home visit	117 (20.0)
2-3 home visits	154 (26.4)
≥4 home visits	185 (31.7)
Involvement of a specialist palliative care team (*n* (%))	
No	355 (60.8)
Yes	229 (39.2)
Days spent at hospital^*∗*^ (median days (IQR))	11 (2; 21)

IQR: Inter Quartile Range.

£: English Pounds.

GCSE: General Certificate of Secondary Education.

GP: general practitioner.

^*∗*^In the last 90 days of the patients' lives.

Not all sums of percentages add up to 100.0% because of round-offs.

**Table 2:** Associations between ≥2 GP face-to-face contacts (including in-office consultations and home visits) and home visits in the last 90 days of life and patient-related socioeconomic and cultural characteristics for those 584 patients, who had a full dataset. The unadjusted prevalence ratios (PRs) and the adjusted PRs for the full model are shown with 95% confidence intervals (95% CIs). The full model includes all variables in the table adjusted for gender, age (continuous variable), primary cancer diagnosis, number of days at hospital (continuous variable), place of death, and involvement of palliative specialist team.

	GP face-to-face contacts				Home visits by the GP			
	(≤1 contact/≥2 contacts)				(No/yes)			
	Unadjusted prevalence ratio (95% CI)	*p* value	Adjusted prevalence ratio (95% CI)	*p* value	Unadjusted prevalence ratio (95% CI)	*p* value	Adjusted prevalence ratio (95% CI)	*p* value
Variables in final model								
Patient's marital status								
Single	1		1		1		1	
Married or cohabiting	0.99 (0.92; 1.07)	0.837	1.05 (0.97; 1.14)	0.189	**0.90 (0.83; 0.98)**	**0.018**	0.97 (0.90; 1.06)	0.525
Having children living at home								
No	1		1		1		1	
Yes	**0.73 (0.55; 0.96)**	**0.024**	0.83 (0.63; 1.09)	0.177	**0.69 (0.50; 0.94)**	**0.019**	1.08 (0.78; 1.48)	0.651
Patient's economic status								
High income (>20000 £/year)	1		1		1		1	
Medium income (10001–20000 £/year)	**1.12 (1.03; 1.23)**	**0.009**	1.04 (0.96; 1.14)	0.329	**1.23 (1.11; 1.36)**	**0.000**	1.09 (0.99; 1.19)	0.071
Low income (0–10000 £/year)	**1.24 (1.11; 1.39)**	**0.000**	**1.18 (1.03; 1.35)**	**0.017**	1.17 (0.97; 1.41)	0.106	1.18 (0.98; 1.42)	0.074
Employment								
Unemployed, social security, student	1				1			
Old age or early retirement pensioner	1.06 (0.95; 1.19)	0.283	Not included because of collinearity with income		**1.30 (1.11; 1.52)**	**0.001**	Not included because of collinearity with income	
Employed or leadership position	**0.83 (0.70; 0.99)**	**0.038**			**0.78 (0.61; 0.99)**	**0.042**		
Ethnicity								
Not immigrant or descendant	1		1		1		1	
Immigrant or descendant	1.10 (0.96; 1.28)	0.175	**1.17 (1.02; 1.35)**	**0.025**	0.72 (0.50; 1.05)	0.090	0.78 (0.55; 1.12)	0.183
Urbanicity, inhabitants in municipality								
10,000 inhabitants	1		1		1		1	
10,000–49,999 inhabitants	0.97 (0.88; 1.06)	0.522	0.97 (0.89; 1.07)	0.576	0.97 (0.87; 1.09)	0.633	1.00 (0.90; 1.12)	0.939
50,000–99,999 inhabitants	0.94 (0.84; 1.04)	0.215	0.92 (0.83; 1.01)	0.095	0.91 (0.80; 1.04)	0.172	0.90 (0.80; 1.02)	0.115
≥100,000 inhabitants	**0.84 (0.76; 0.93)**	**0.001**	**0.85 (0.77; 0.95)**	**0.003**	**0.86** **(0.77; 0.96)**	**0.008**	**0.89 (0.80; 0.99)**	**0.037**

The final model was adjusted for the following variables								
Gender of patient								
Male	1		1		1		1	
Female	1.00 (0.92; 1.07)	0.917	0.98 (0.90; 1.07)	0.635	1.02 (0.93; 1.11)	0.719	0.96 (0.87; 1.06)	0.461
Age of patient (continuous variable)	**1.01 (1.00; 1.01)**	**0.000**	**1.00 (1.00; 1.01)**	**0.020**	**1.01 (1.01; 1.02)**	**0.000**	**1.01 (1.01; 1.02)**	**0.000**
Primary cancer diagnosis								
Bronchus/lung	1		1		1		1	
Colon/rectum	0.91 (0.81; 1.02)	0.117	0.91 (0.82; 1.02)	0.124	0.87 (0.75; 1.00)	0.056	**0.83 (0.73; 0.95)**	**0.008**
Prostate	0.90 (0.79; 1.02)	0.086	**0.85 (0.74; 0.96)**	**0.010**	1.05 (0.94; 1.17)	0.385	0.94 (0.84; 1.05)	0.276
Breast	0.89 (0.77; 1.02)	0.088	0.91 (0.79; 1.04)	0.158	**0.83 (0.69; 0.98)**	**0.032**	0.86 (0.71; 1.03)	0.109
Other	**0.88 (0.81; 0.97)**	**0.006**	**0.90 (0.81; 0.99)**	**0.031**	**0.87 (0.78; 0.96)**	**0.009**	**0**.**88 (0.79; 0.97)**	**0.010**
Days spent at hospital in the last 90 days of the patient's life (continuous variable)	**0.99 (0.99; 1.00)**	**0.000**	**0.99 (0.99; 1.00)**	**0.011**	**0.99 (0.99; 1.00)**	**0.000**	1.00 (0.99; 1.00)	0.199
Place of death								
Institutional death (Hospital/hospice)	1		1		1		1	
Home/nursing home death	**1.28 (1.17; 1.39)**	**0.000**	**1.19 (1.09; 1.29)**	**0.000**	**1.47 (1.33; 2.63)**	**0.000**	**1.33 (1.21; 1.47)**	**0.000**
Involvement of a specialist team								
Yes	1		1		1		1	
No	1.05 (0.98; 1.13)	0.189	1.06 (0.99; 1.14)	0.105	1.04 (0.95; 1.13)	0.384	**1.10 (1.01; 1.20)**	**0.029**

GP: general practitioner.

£: English Pounds.

Note: significant correlations with a *p* value < 0.05 are in bold text.
